# Orbital infarction syndrome following mechanical thrombectomy in internal carotid artery occlusion: a case report

**DOI:** 10.1186/s13256-024-04419-0

**Published:** 2024-03-08

**Authors:** Binh Q. Tran, Lanh C. Nguyen, Tinh T. Trinh, An T. T. Vo, Minh V. Do, Minh Q. Tran, Khanh C. Tran, Loan H. T. Tran, Duc T. Ha

**Affiliations:** 1 Department of Stroke Care, Can Tho Central General Hospital, 315. Nguyen Van Linh Street, Ninh Kieu District, Can Tho City, Vietnam; 2 Department of Neurosurgery, Can Tho Central General Hospital, Can Tho City, Vietnam; 3 Department of Diagnostic Imaging, Can Tho Central General Hospital, Can Tho City, Vietnam; 4 Phuong Chau International Hospital, Can Tho City, Vietnam

**Keywords:** Orbital ischemic syndrome, Case report, Mechanical thrombectomy, Ischemic stroke, Internal carotid artery

## Abstract

**Background:**

Orbital ischemic syndrome is a rare entity. The clinical signs typically demonstrate acute loss of visual acuity, chemosis, proptosis, ptosis, and total ophthalmoparesis. We report a case of a man who suffered an acute internal carotid artery occlusion and developed orbital ischemic syndrome after a mechanical thrombectomy.

**Case presentation:**

A 57-year-old Vietnamese (Kinh ethnicity) man was brought to the emergency room with complaints of a speech disturbance, facial palsy, and severe weakness of the left arm and leg, which had started 4 hours earlier, after waking up. The National Institutes of Health Stroke Scale 12 (NIHSS 12) revealed the neurological score at admission. A head computed tomography scan showed no intracranial bleeding and an Alberta Stroke Program Early Computed Tomography Score (ASPECTS) of 8 on the right brain. Computed tomography angiography showed an occlusion of the right internal carotid artery. After that, a mechanical thrombectomy was performed, and the internal carotid artery was completely reperfused. After 10 hours, he experienced orbital pain, proptosis, ptosis, chemosis, and ophthalmoplegia of the right orbit. He also had acute loss of visual acuity, and fundoscopic examination revealed papilledema, no retinal hemorrhage, and no bruit in orbital auscultation. Intraocular pressure in the right eye was measured at 50.5 mmHg. Computed tomography angiography showed no carotid–cavernous fistula, but slight enlargement of the right medial and lateral rectus muscles. He was treated with steroids and hyperosmolar agents and recovered 7 days later, but had persistent loss of visual acuity in the right eye.

**Conclusion:**

Orbital ischemic syndrome is a rare complication after mechanical thrombectomy treatment in acute ischemic stroke that can lead to loss of visual acuity.

**Supplementary Information:**

The online version contains supplementary material available at 10.1186/s13256-024-04419-0.

## Background

Orbital ischemic syndrome (OIS) is the ischemia of all intraorbital and intraocular structures, including the optic nerve, extraocular muscles, and orbital [1]. It was first described by Vergez in 1959 [2], who noted that patients suffering from OIS will demonstrate acute loss of visual acuity, chemosis, proptosis, ptosis, and total ophthalmoparesis. Even though this is a rare condition, there are many etiologies reported in the literature, such as infectious and inflammatory diseases, hypoperfusion from common carotid artery occlusion, and invasive procedures, such as aneurysm surgery or endovascular mechanical thrombectomy (MT) [3–7]. Recently, OIS has been considered a complication of MT [8]. We report a case of OIS after the MT of the acute internal carotid artery (ICA) occlusion stroke.

## Case presentation

### Patient information and clinical findings

A 57-year-old Vietnamese (Kinh ethnicity) man presented with a speech disturbance, facial palsy, and severe weakness of the left arm and leg after awakening; the average time from symptom onset to presentation was 6.5 h. His medical history included biological aortic valve replacement 7 months ago, pacemaker implantation, and hypertension. On admission, a neurological exam revealed dysarthria, left-sided hemiparesis, and head deviation to the right side; the National Institutes of Health Stroke Scale 12 (NIHSS 12) revealed the neurological score.

### Diagnostic assessment

**Fig. 1:**
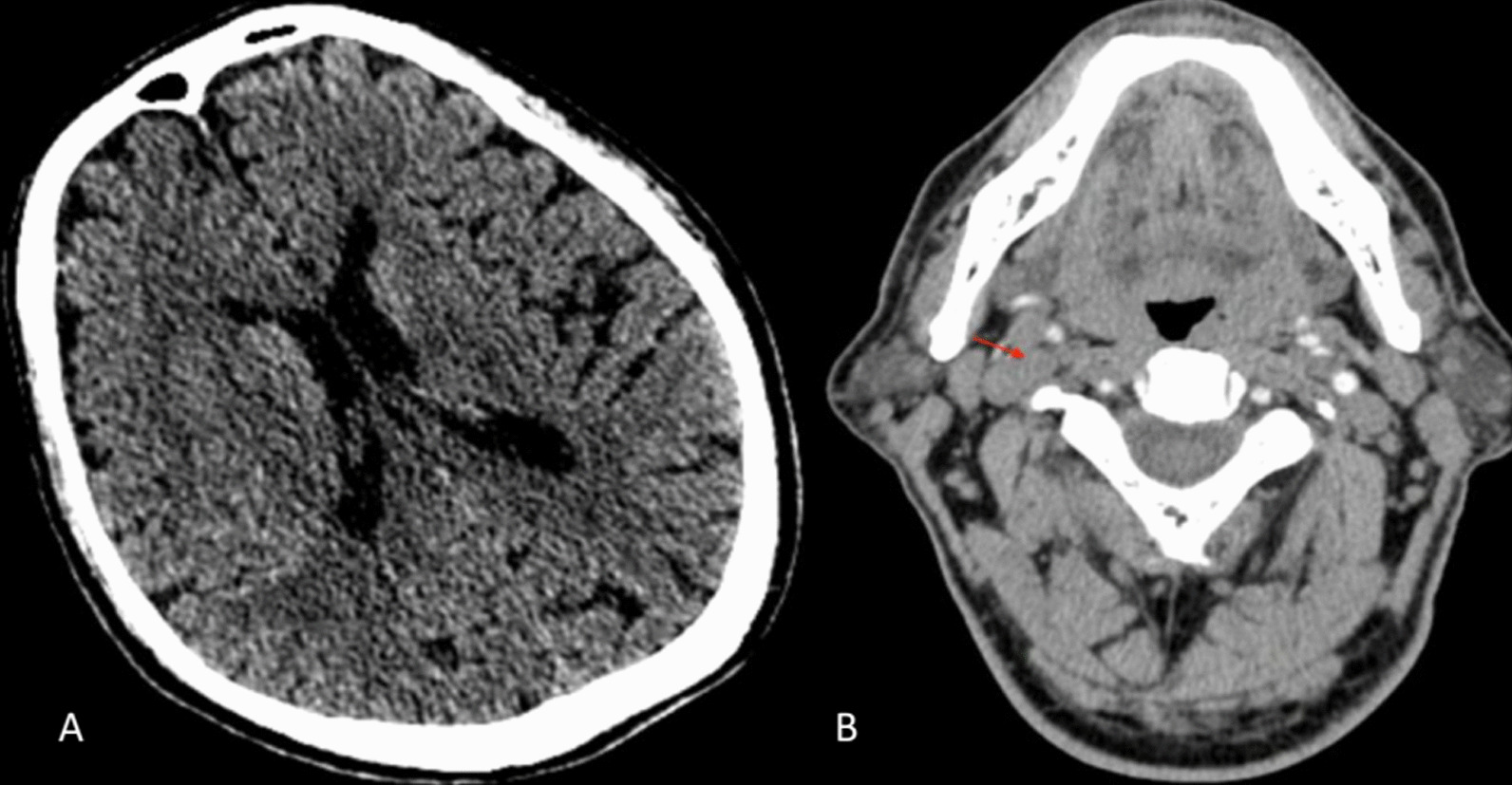
Noncontrast computed tomography (NCCT) showing early ischemic lesions on the right temporal lobe (**A**). CTA showed right ICA occlusion (red arrow) (**B**)

An urgent non-contrast computed tomography of the brain showed no hemorrhage, with an Alberta Stroke Program Early Computed Tomography Score (ASPECTS) of 8 on the right brain (Fig. 1). After that, computed tomography angiography (CTA) showed right proximal ICA occlusion (Fig. 1). He was then transferred to the angiography suite for mechanical thrombectomy.

### Therapeutic intervention

**Fig. 2:**
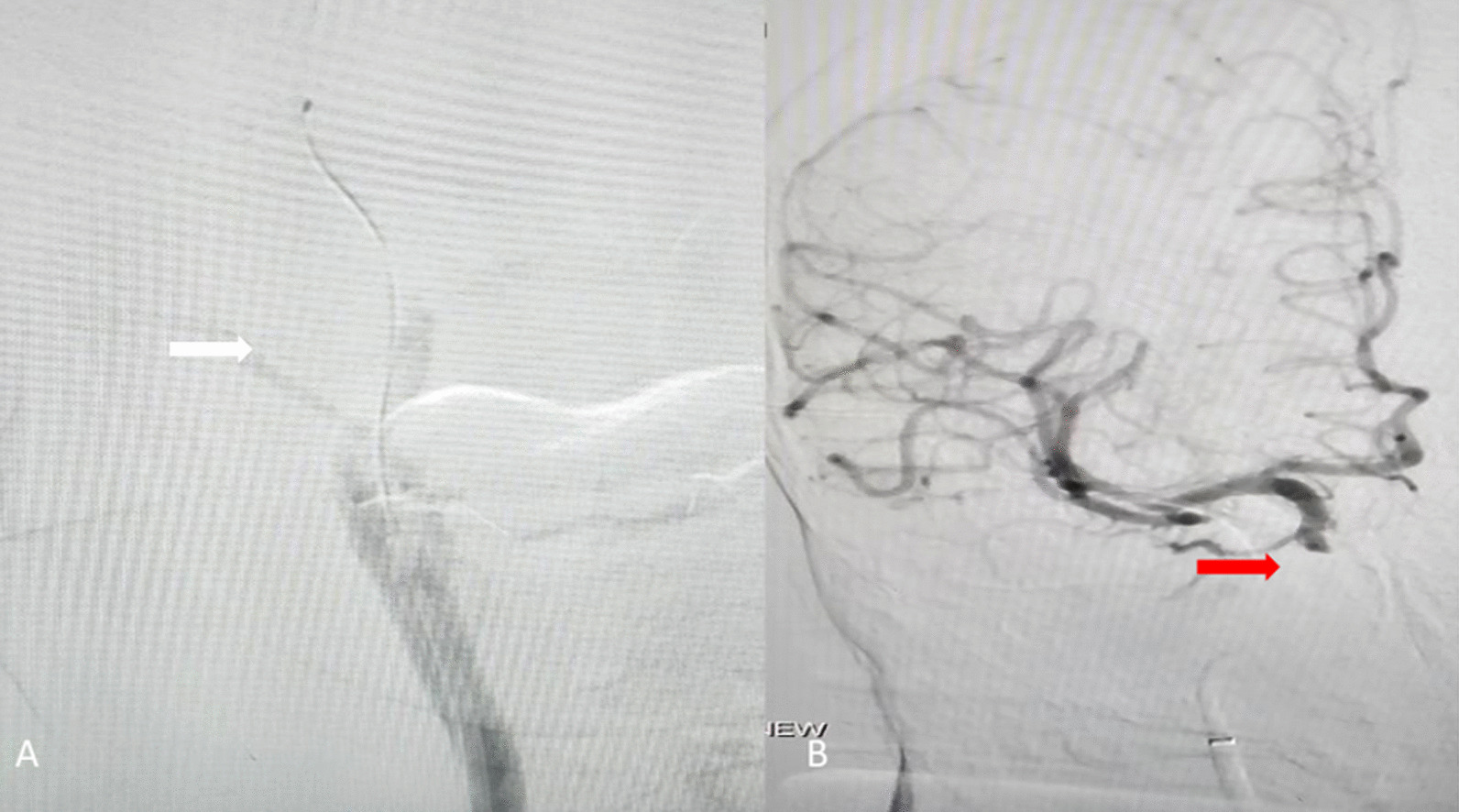
Occlusion of the cavernous right ICA (white arrow) (**A**). Following the third pass, the ICA was recanalized (red arrow) (**B**)

**Fig. 3:**
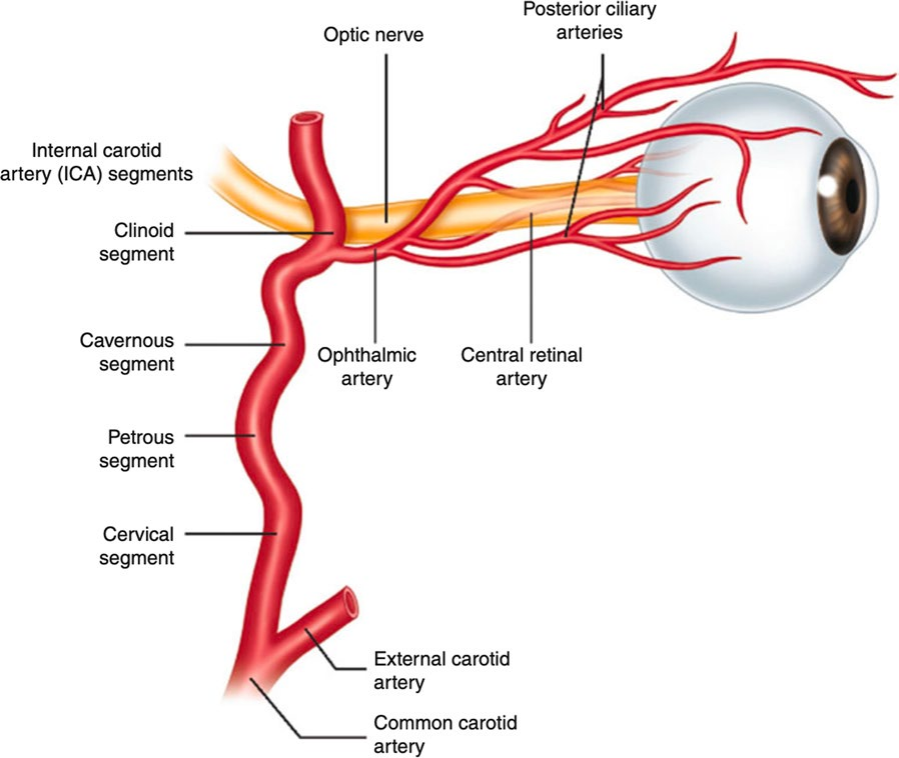
Internal carotid artery and ophthalmic artery vascular anatomy

Transfemoral cerebral angiography confirmed an occlusion at the cavernous segment of the right ICA (Fig. 2). The clot was removed after the third pass using a 4 × 20 mm stent retriever. The final angiogram of the right ICA showed modified Thrombolysis in Cerebral Ischemia 3 (mTICI 3) (Fig. 2) and without occlusion of the right ophthalmic artery. Postoperatively, the NIHSS score improved by 7/42. In the following hours, blood pressure was 120/60 mmHg. Internal carotid artery and ophthalmic artery vascular anatomy are shown in Fig. 3 [9].

### Follow-up and outcomes

After 10 hours, he complained of right orbital pain (Fig. 2A). Clinical examination revealed acute visual loss, ptosis, proptosis, chemosis, and complete ophthalmoplegia. However, we did not discover any audible bruit in the orbit. The funduscopic examination showed papilledema with no retinal hemorrhage. Intraocular pressure in the right eye was measured at 50.5 mmHg.

**Fig. 4:**
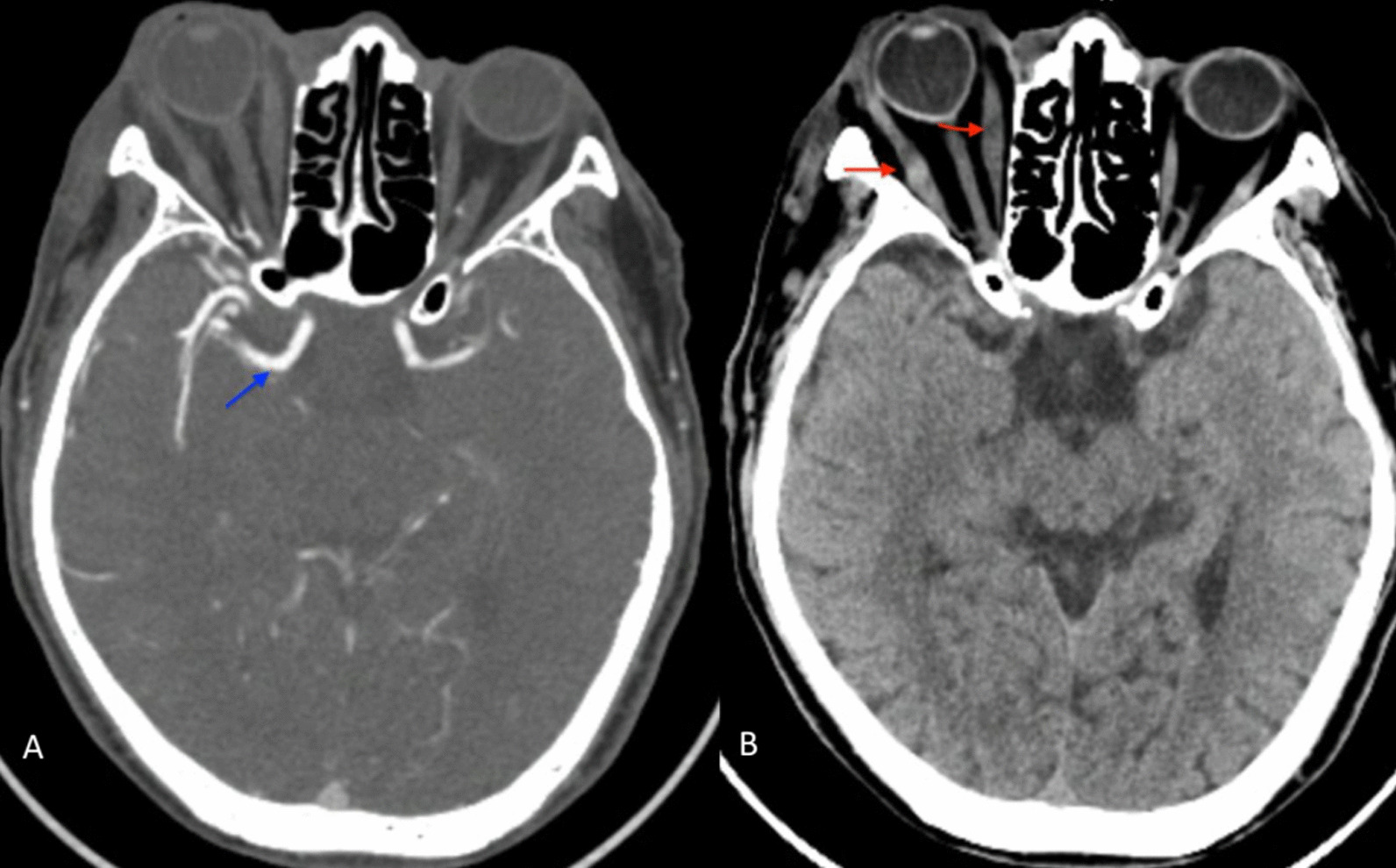
CTA after MT revealing no signs of carotid–cavernous fistula (blue arrow) (**A**). Slight enlargement of the right medial and lateral rectus muscles (red arrows) (**B**)

To rule out a possible carotid–cavernous fistula, CTA was performed, and the result showed no sign of carotid–cavernous fistula, but slight enlargement of the right medial and lateral rectus muscles (Fig. 4).

**Fig. 5:**
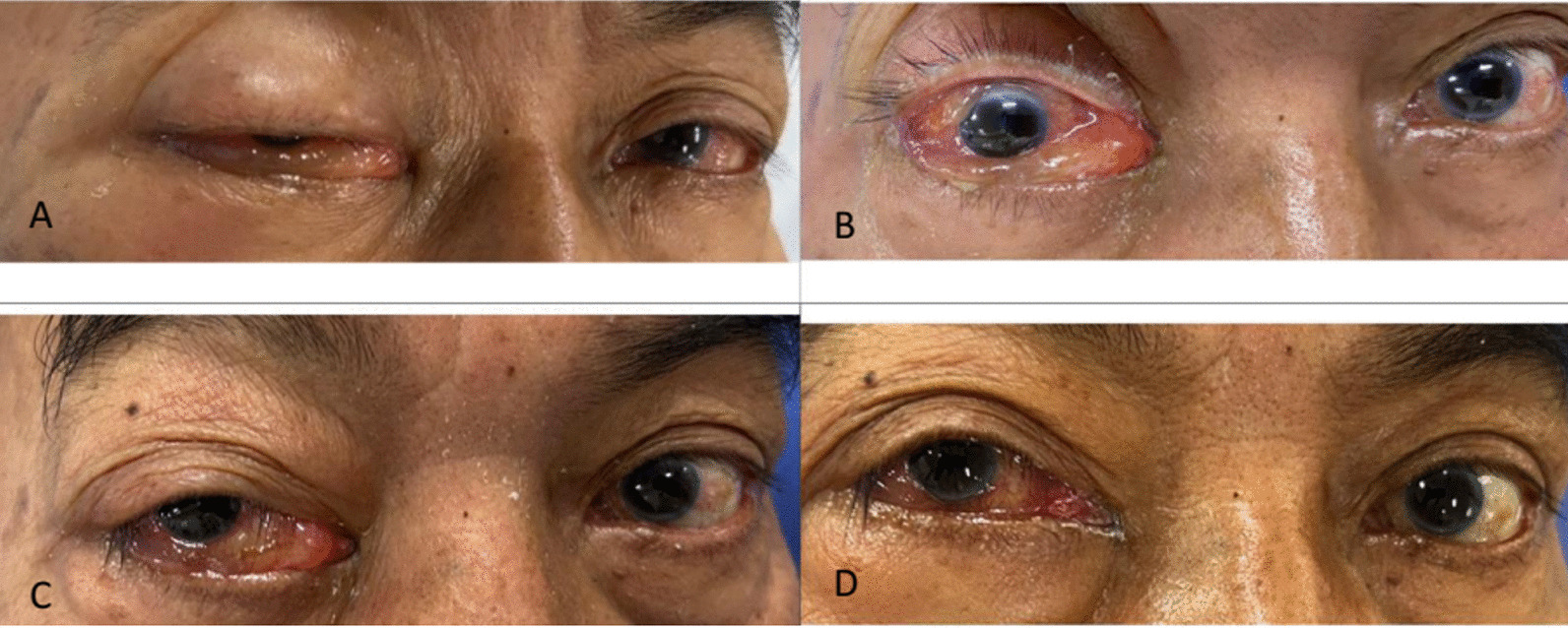
Clinical signs of the patient after mechanical thrombectomy with chemosis, ptosis, and visual loss (**A**). Ptosis and chemosis improved 2 days, 5 days, 7 days later (**B**, **C**, **D**)

He was treated with steroids and drugs attributed to reducing intraocular pressure, such as mannitol and acetazolamide. His ophthalmological symptoms improved after 7 days (Fig. 5C, D), and intraocular pressure in the right eye ranged between 17 mmHg and 20 mmHg, but the left had loss of visual acuity. The patient made a meaningful neurological recovery with an NIHSS score of 6 and was discharged home 7 days after the initial episode (Additional file 1).

## Discussion and conclusion

We present a rare case of OIS following MT for treatment of right ICA occlusion stroke. OIS is rare; numerous cases have recently been reported [3–6, 8, 10, 11]. In terms of the anatomy of the ophthalmic artery (OA), OA typically arises from ICA, but some variations change the origin of OA. OA can arise from a middle meningeal artery, intracavernous ICA, supraclinoid ICA, anterior cerebral artery, or external carotid artery. Regarding the blood supply to the orbit, the primary source of blood supply comes from the OA. However, the external carotid artery contributes little to the orbit blood supply through the infraorbital artery and orbital branch of the middle meningeal artery [12, 13]. Thanks to a wide range of variations of OA and various anastomoses between the OA and many branches of the external carotid artery [12], the occlusion of the ICA or the OA hardly ever results in OIS.

Patients have symptoms such as proptosis, chemosis, ophthalmoplegia, and painful visual loss after MT that make incorrect diagnoses with carotid–cavernous fistula (CCF) if clinical signs are relied upon, primarily because both entities have the same clinical symptoms, though CCF is the more serious. A CCF complication should thus be suspected at first. It is also quite challenging to handle it well if not recognized quickly. Here are some tips that can help to differentiate them. We did not see corkscrew vessels in conjunctiva and episcleral without a bruit in orbital auscultation. CTA showed no signs of CCF; therefore, a CCF diagnosis was ruled out. In addition, CTA also revealed slight enlargement of the right medial and lateral rectus muscles, which would help in defining right orbital inflammation. Furthermore, high intraocular pressure in the right eye leads to orbital ischemia.

In our case, the patient suffered an acute ischemic stroke due to acute occlusion of the cavernous segment of the ICA. The mechanism of orbital infarction in our patient was more likely due to the direct effect of the mechanical forces exerted by the stent retriever and other devices that facilitate the release of inflammatory mediators [14, 15]. The implication is that it would result in rising intraocular pressure, thereby leading to hypoperfusion of the OA to the orbit. As a result, the hypoperfusion of the orbit induces OIS. Although the patient was treated intensively with steroids and hyperosmolar agents, the visual acuity in his right eye was lost. The mechanism causing OIS was explained similarly in previous mechanical thrombectomy clinical cases [5, 8]

This case provides us with some outstanding merits. Firstly, clinicians must raise awareness of monitoring stroke cases following MT, which developed a proptosis to recognize the complications of sporadic cases such as the OIS and the CCF. Secondly, the appropriate treatment of OIS was still challenging when a medical treatment like steroids and hyperosmolar agents was not proven. Therefore, further studies are needed to define the therapeutic approach in such cases.

OIS after MT to treat acute ischemic stroke is rare and must be observed thoroughly to approach a proper treatment, despite mostly leaving poor clinical outcomes. OIS should be considered a complication after MT therapy when patients have clinical evidence of orbital ischemia, such as visual loss, proptosis, chemosis, ophthalmoparesis, and imagiologic evidence consistent with this disorder after mechanical thrombectomy.

### Supplementary Information

**Additional file 1.** National Institutes of Health Stroke Scale (NIHSS).

## Data Availability

Not applicable.

## Abbreviations

ASPECTS: Alberta Stroke Program Early Computed Tomography Score
CCF: Carotid–cavernous fistula
CT: Computed tomography
CTA: Computed tomography angiography
ICA: Internal carotid artery
MT: Mechanical thrombectomy
NIHSS: National Institutes of Health Stroke Scale
OA: Ophthalmic artery
OIS: Orbital ischemic syndrome
